# HPV genotypic spectrum in Jilin province, China, where non-vaccine-covered HPV53 and 51 are prevalent, exhibits a bimodal age-specific pattern

**DOI:** 10.1371/journal.pone.0230640

**Published:** 2020-03-24

**Authors:** Sijia Hao, Chunyan Wang, Siwen Liu, Jiaxue He, Yanfang Jiang

**Affiliations:** Key Laboratory of Organ Regeneration & Transplantation of the Ministry of Education, Genetic Diagnosis Center, The First Hospital of Jilin University, Changchun, China; Penn State University School of Medicine, UNITED STATES

## Abstract

**Background:**

Human papillomavirus (HPV), the most common sexually transmitted disease, is involved in a series of other diseases. The persistent infection of high-risk HPVs (HR-HPVs) is considered to be the causative agent of cervical cancer, and it is related to noncervical cancers. The present study aims to estimate the HPV prevalence and genotype distribution in Jilin province, China, to guide HPV-related cervical cancer screening and HPV vaccination.

**Methods:**

From October 2017 to September 2019, 21,282 samples (634 male and 20,648 female) were collected for HPV infection detection using an HPV genotyping panel. The age-related HPV prevalence and morbidity of HPV-based disease and HPV prevalence associated with specific diseases were analyzed.

**Results:**

A total of 7095 (34.4%) positive for HPV infection of 20648 women, and 164 (25.8%) positive of 634 men. The HPV prevalence among women exhibited a bimodal pattern, with a peak in young group and a second peak in old group, with increased severity of cervical lesions. HPV16 (7.8%), HPV52 (5.8%), HPV58 (5.0%), HPV53 (3.4%), and HPV51 (3.0%) were the most prevalent genotypes among women, and HPV6 (6.0%), HPV11 (5.7%), HPV16 (3.6%), HPV18 (2.7%), and HPV51 (3.0%) were prevalent among men. Non-vaccine-covered HPV53 and 51 were found in 6.3% of HPV infection and 8.9% of cervical cancer in Jilin province. Furthermore, 45.5% of females and 28.6% of males with genital warts were infected with HR-HPV genotypes.

**Conclusion:**

The HPV genotypic spectrum in Jilin province, where non-vaccine-covered HPV53 and 51 were prevalent, exhibited an age- and cervical lesion-specific pattern, which provides guidance for HPV vaccination and cervical cancer screening. HPV infection in men and benign hyper-proliferative lesions should not be neglected.

## Introduction

Human papillomavirus (HPV) is the most common sexually transmitted disease worldwide, with a prevalence of 70 million cases and an incidence of 14 million new transmissions are reported annually [1]. HPV infection exhibits a distinct tropism for mucosal or cutaneous squamous epithelia, and the position where cancer derives depends on the site of HPV invasion. HPV infection is involved in a series of diseases of the vagina, vulva, penis, and anus, as well as anogenital warts and recurrent respiratory papillomatosis [2]. In 2018, there were estimated to be 570,000 new cases of cervical cancer and 311,000 deaths related to cervical cancer, representing 6.6% of all female cancers; cervical cancer was ranked as the fourth most frequently diagnosed cancer and the fourth leading cause of cancer death in women [2–4].

HPVs are classified as high-risk HPVs (HR-HPVs), intermediate-risk HPVs (IR-HPVs) and low-risk HPVs (LR-HPVs) based on their association with cervical cancer [5], and these viruses ubiquitously reside on the skin of humans without signs of symptomatic lesions and compose part of the normal microbial skin flora. Most HPV infections remain asymptomatic and may spontaneously regress, but some infections may further develop into cervical intraepithelial neoplasia (CIN) and cervical carcinoma [6].

The persistent infection of HR-HPVs is considered to be the causative agent of cervical cancer [7]. The International Agency for Research on Cancer (IARC) indicated HR-HPVs caused 95% of all cervical cancers [8]. HPV16 and HPV18 are detected in approximately 70% of cervical cancers and approximately 86% to 95% of HPV-associated noncervical cancers, such as anal cancers, oropharyngeal cancers, vaginal cancers, vulvar cancers and penile cancers [9].

In contrast, LR-HPVs are primarily linked to benign hyper-proliferative lesions, such as common warts and genital warts, and these viruses are not a frequent cause of malignant carcinoma [3]. Recalcitrant genital warts and ubiquitous common warts cannot be reliably eradicated, and few treatment strategies were advanced. Because of the low risk of driving neoplasia and cancer progression, the available data about the prevalence of LR-HPVs are still insufficient.

The prevalence and types of HPV varies between nations and regions and show a strong association with the level of development. Cervical cancer is the most commonly diagnosed cancer and the leading cause of cancer death in countries within a low ranking on the Human Development Index (HDI), especially those in Sub-Saharan Africa and South-Eastern Asia [3, 10]. Considering its large population with geographical and socioeconomic inequities, China has a large burden of cervical cancer and important disparities among different regions [11]. Because of the widespread screening of cervical cancer, the incidence in America has decreased more than 50% in the past 30 years [12, 13]. Fully understanding the epidemiological characteristics of HPV infection is of great importance to the development of prevention and control strategies for HPV.

In this study, we investigate the age-related HPV prevalence and morbidity of HPV-based disease. We elucidated the spectrum of the prevalence and genotype distribution of human papillomaviruses in Jilin province, China, and the HPV prevalence according to cervical lesions and HPV prevalence associated with common warts and genital warts.

## Materials and methods

### Ethics statement

The Ethics Committee of First Hospital of Jilin University approved this project. All of the samples and data were collected after written informed consent was provided by the participants. The management and publication of patient information in this research was strictly in accordance with the Declaration of Helsinki, including the confidentiality and anonymity.

### Study population

A retrospective database review was conducted to identify all HPV genotyping results reported from October 2017 to September 2019. All the HPV tests were performed at the genetic diagnosis center. Samples for HPV genotyping were collected from the First Hospital of Jilin University, including gynaecology, andrology, dermatology and physical examination center.

21,282 samples were collected from 634 male individuals and 20,648 female individuals. The HPV genotyping results and relevant clinical information were all recorded, including age and clinical history available in database. All patients reported no previous diagnosis of HIV infection. The study of HPV prevalence associated with cervical lesions demonstrated that 1156 patients were normal gynecological outpatients, 1498 were diagnosed with cervicitis. Of the 385 patients diagnosed with CIN, 51 without specific category, 126 were CIN-I, 78 were CIN-II and 130 were CIN-III. 101 patients were diagnosed with cervical cancer.

Diagnosis of cervical disease were confirmed via cytological diagnosis, colposcopy and cervical biopsy. Cervicitis were characterized by vaginal discharge, vaginal bleeding, cervical erythema, friability, erosion, and edema, but without intraepithelial lesion or malignancy. The histological type of cervical cancer includes squamous cell carcinoma, adenocarcinoma, adenosquamous cell carcinoma.

Common warts present as papules, round or polygon, rough surface, keratinization, hard, yellowish gray. They occur at many sites, but often on the back of hands. Genital warts, also called condyloma acuminatum, present as papules, nodules or soft, filiform, pinkish, sessile or pedunculated growths.

### DNA extraction and HPV genotyping

Cervical cells were collected with a cytobrush from ectocervix and endocervix of the uterus by cervical scrapings. And epithelial cells from common warts and genital warts were collected with a cytobrush by epithelial brushing. For patients with multiple warts, all visible lesions were brushed. The samples were stored at 4°C in the standard media provided with the panel for DNA extraction. DNA isolation and purification were conducted according to the manufacturer’s instructions (Hybribio Limited, Chaozhou, China).

All HPV tests were performed with an HPV genotyping panel (polymerase chain reaction (PCR)-reverse dot blot hybridization method (Hybribio Limited, Chaozhou, China), which identified 14 HR-HPV types (16, 18, 31, 33, 35, 39, 45, 51, 52, 56, 58, 59, 66 and 68), 1 IR-HPV type (53), and 6 LR-HPV types (6, 11, 42, 43, 44, and 81). HPV DNA was extracted, amplified, and genotyped according to the manufacturers’ protocol. The PCR program consisted of an initial step at 95 °C for 9 min, 40 cycles of 95 °C for 20 s, 55 °C for 30 s, 72 °C for 30 s, and a final extension at 72 °C for 5 min. The HPV type-specific probes were immobilized on nylon membranes, which were used for reverse-blot hybridization and detection of all HPV genotypes in a single assay. Sterile water and specimens with known HPV genotypes were used as the negative and positive controls, respectively.

### Statistical analysis

Data analysis was performed using the statistical software packages Graph Pad Prism 5 (Graph Pad Software, La Jolla, CA) and SPSS 25.0 (IBM, Armonk, NY, USA). The chi-squared test was used for statistical analysis between two groups. P-values were two-sided, and differences were considered statistically significant at p < 0.05.

## Results

### Age-related HPV prevalence

From October 2017 to September 2019, 21,282 samples were collected from 634 male individuals and 20,648 female individuals, for HPV infection detection at the genetic diagnosis center. With 7095 (34.4%) positive of 20648 women, 164 (25.8%) positive of 634 men. All samples were divided into 9 age groups: ≤ 24 years old, 25 to 29 years old, 30 to 34 years old, 35 to 39 years old, 40 to 44 years old, 45 to 49 years old, 50 to 54 years old, 55 to 59 years old, and ≥ 60 years old.

The HPV infection prevalence among women were 51.0%, 39.3%, 35.0%, 31.4%, 31.3%, 30.9%, 32.8%, 37.8%, 35.3%, respectively, showed an age-related prevalence (p < 0.001). (Table 1) HPV16, 18, 39, 51, 53, 56, 58, 59, 6, 11 43 (p < 0.001) and HPV31, 52, 66, 81 (p = 0.001) showed an age-related prevalence. The p = 0.033 for HPV45 also showed an age-related prevalence. The incidence of HPV6 and HPV11 decreased with age. The highest infection rate of most HPV genotypes, except HPV35, 52, 53, 58, 42, 44 and 81, was found in the ≤ 24 years old group.

**Table 1:** Overall age-related HPV prevalence among women.

	≤24(%)	25-29(%)	30-34(%)	35-39(%)	40-44(%)	45-49(%)	50-54(%)	55-59(%)	≥60(%)	Total prevalence
N	926	2141	3138	3490	3008	3125	2105	1423	1292	
Prevalence	51.0	39.3	35.0	31.4	31.3	30.8	32.8	37.8	35.3	
**16**	12.3	7.1	8.2	7.4	6.3	6.9	7.7	10.3	8.4	74.7
**18**	5.9	3.1	2.6	2.3	1.5	1.9	1.9	3.0	2.2	24.4
**31**	3.2	2.5	2.3	1.8	2.0	1.3	1.8	2.5	3.1	20.5
**33**	2.6	2.4	1.7	1.9	1.5	2.0	1.6	2.3	2.2	18.3
**35**	0.8	0.9	0.5	0.5	0.8	0.8	0.7	0.8	0.9	6.7
**39**	5.4	3.1	3.4	2.4	2.8	2.1	2.6	2.2	2.6	26.7
**45**	1.6	0.7	0.6	0.7	0.5	0.6	0.6	1.1	0.6	7.0
**51**	6.6	4.1	2.5	2.7	2.7	2.4	3.3	3.1	2.5	29.8
**52**	7.1	6.5	6.3	4.7	5.9	5.2	4.8	7.2	6.7	54.6
**56**	3.7	2.0	1.3	1.5	1.8	1.5	2.3	2.5	2.5	18.9
**58**	7.3	6.0	4.6	4.0	4.5	4.8	4.5	5.6	7.6	48.9
**59**	3.2	2.0	1.1	1.3	1.3	1.0	1.1	1.5	1.5	13.9
**66**	3.2	1.3	2.1	1.8	1.9	1.6	1.9	2.3	3.2	19.4
**68**	2.8	2.0	2.2	1.7	1.6	1.8	1.9	1.4	1.5	17.0
**53**	3.1	4.1	3.4	2.8	2.4	2.8	4.4	4.6	4.3	31.9
**6**	9.0	3.2	2.7	1.4	1.1	1.1	1.1	1.6	1.5	22.6
**11**	8.9	2.6	1.7	1.1	1.2	0.8	1.3	0.9	1.3	19.7
**42**	0.8	0.7	0.3	0.4	0.4	0.5	0.8	0.6	0.8	5.1
**43**	1.3	0.3	0.2	0.1	0.3	0.3	0.3	0.1	0.3	3.3
**44**	0.5	0.5	0.4	0.5	0.8	1.0	0.7	0.8	0.6	5.8
**81**	2.4	2.0	1.4	1.8	1.7	1.8	2.5	1.4	3.3	18.3

The overall prevalence of HR-HPV genotypes (HPV16, 18, 31, 33, 35, 39, 45, 51, 52, 56, 58, 59, 66, and 68), IR-HPV genotypes (HPV53) and LR-HPV genotypes (HPV6, 11, 42, 43, 44, and 81) according to age among women.

**Fig 1:**
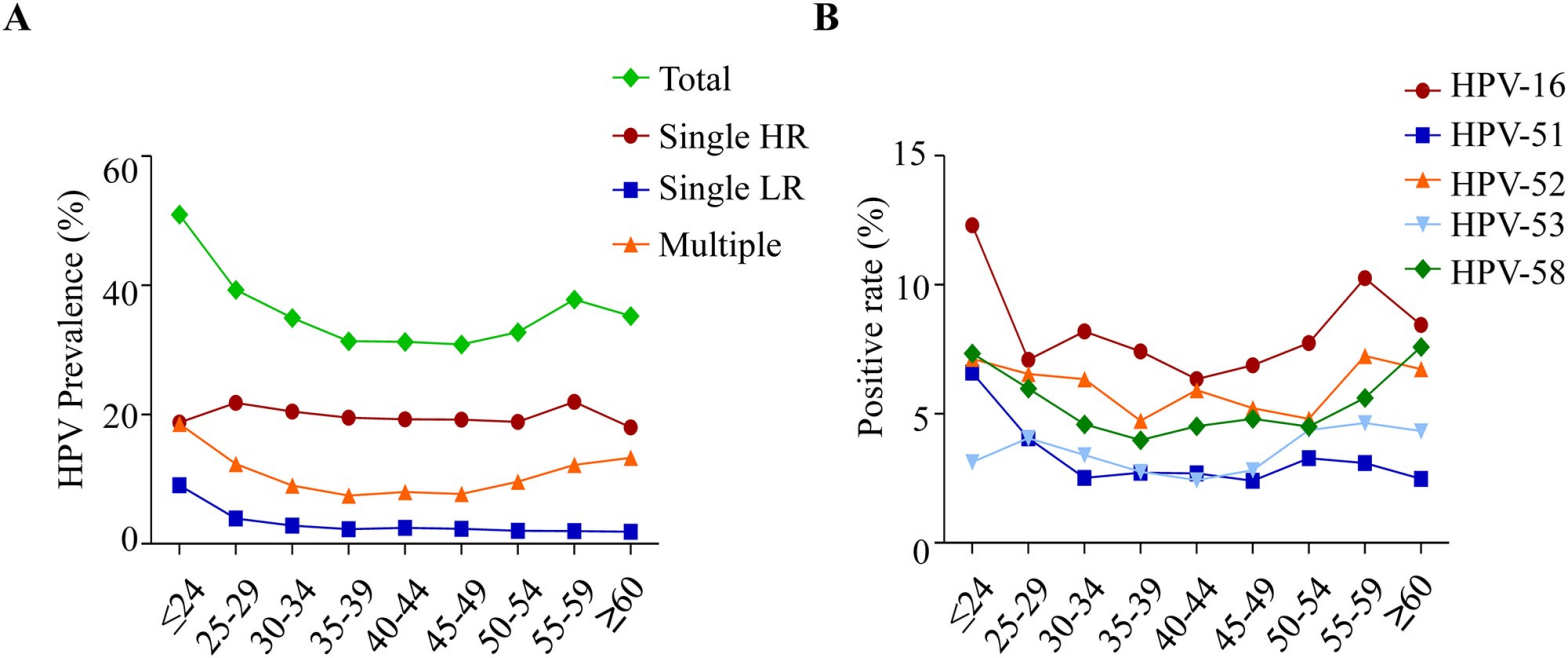
Overall age-related HPV prevalence among women. (A) The prevalence of total HPV, single HR-HPV, single LR-HPV and multiple HPV infections according to age. (B) The prevalence of the age-related 5 most prevalent genotypes HPV16, 51, 52, 53 and 58.

The prevalence of total HPV, single HR-HPV, single LR-HPV and multiple HPV infections according to age is shown. (Fig 1A) HR-HPV infection (p = 0.037), LR-HPV infection (p < 0.001) and multiple HPV infections (p < 0.001) showed an age-related prevalence. A bimodal pattern was shown in the prevalence of total HPV infection, single HR-HPV infection and multiple HPV infections, with a peak at younger group and a second peak at elderly group. The prevalence of single LR-HPV infection decreased with age.

The prevalence of the 5 most prevalent age-related genotypes HPV16, 51, 52, 53, and 58 is shown in Fig 1B. The peak value of HPV16 appeared at 30 to 34 years old and 55 to 59 years old. The peak value of HPV52 appeared at 30 to 34 years old, 40 to 44 years old and 55 to 59 years old. The prevalence of HPV58 decreased with age from 25 to 39 then increased with age and became the 2nd most common genotype at ≥ 60 years old. The prevalence of HPV51 decreased with age before 35 years old, then maintained a stable level.

The HPV infection prevalence among men were 32.6%, 23.4%, 26. 9%, 25.3%, 16.7%, 29.3%, 28.2%, 27.3%, 29.4%, respectively. No obvious difference was observed in the distribution of HPV infection with age among men (p = 0.63). (Table 2) Therefore, it’s no sense to analyze the relationship between the HPV prevalence and the morbidity of HPV-based disease with age.

**Table 2:** Overall age-related HPV prevalence among men.

	≤24(%)	25-29(%)	30-34(%)	35-39(%)	40-44(%)	45-49(%)	50-54(%)	55-59(%)	≥60(%)	Total prevalence
N	92	158	119	91	66	41	39	11	17	
Prevalence	32.6	23.4	26.9	25.3	16.7	29.3	28.2	27.3	29.4	
**16**	5.4	3.8	3.4	2.2	1.5	7.3	5.1	0.0	0.0	28.8
**18**	5.4	3.2	2.5	1.1	1.5	0.0	5.1	0.0	0.0	18.9
**31**	4.3	0.6	0.0	0.0	0.0	0.0	0.0	0.0	0.0	5.0
**33**	1.1	0.6	1.7	0.0	0.0	2.4	0.0	0.0	0.0	5.8
**35**	1.1	0.6	0.8	0.0	0.0	2.4	0.0	0.0	0.0	5.0
**39**	3.3	0.6	0.8	2.2	1.5	0.0	7.7	0.0	0.0	16.1
**45**	0.0	0.6	0.8	0.0	0.0	0.0	0.0	0.0	0.0	1.5
**51**	4.3	1.9	2.5	4.4	3.0	0.0	0.0	9.1	11.8	37.0
**52**	1.1	1.3	2.5	0.0	1.5	2.4	5.1	0.0	0.0	14.0
**56**	0.0	0.0	0.0	0.0	0.0	0.0	0.0	0.0	0.0	0.0
**58**	1.1	0.6	1.7	1.1	0.0	2.4	2.6	0.0	0.0	9.5
**59**	0.0	0.6	0.0	0.0	0.0	0.0	0.0	9.1	0.0	9.7
**66**	3.3	0.6	0.8	4.4	1.5	2.4	0.0	0.0	5.9	19.0
**68**	0.0	0.6	0.8	2.2	1.5	0.0	0.0	0.0	0.0	5.2
**53**	1.1	1.9	1.7	3.3	1.5	2.4	2.6	0.0	5.9	20.4
**6**	6.5	6.3	5.9	7.7	4.5	9.8	2.6	0.0	5.9	49.2
**11**	9.8	6.3	8.4	3.3	0.0	2.4	0.0	18.2	5.9	54.3
**42**	0.0	1.3	1.7	0.0	0.0	2.4	2.6	0.0	0.0	7.9
**43**	0.0	0.6	0.0	1.1	3.0	0.0	0.0	0.0	5.9	10.6
**44**	0.0	2.5	0.0	0.0	0.0	2.4	0.0	0.0	0.0	5.0
**81**	1.1	0.6	0.0	0.0	0.0	0.0	0.0	0.0	0.0	1.7

The overall prevalence of HR-HPV genotypes (HPV16, 18, 31, 33, 35, 39, 45, 51, 52, 56, 58, 59, 66, and 68), IR-HPV genotypes (HPV53) and LR-HPV genotypes (HPV6, 11, 42, 43, 44, and 81) according to age among women.

### Age-related morbidity of HPV-based disease

**Fig 2:**
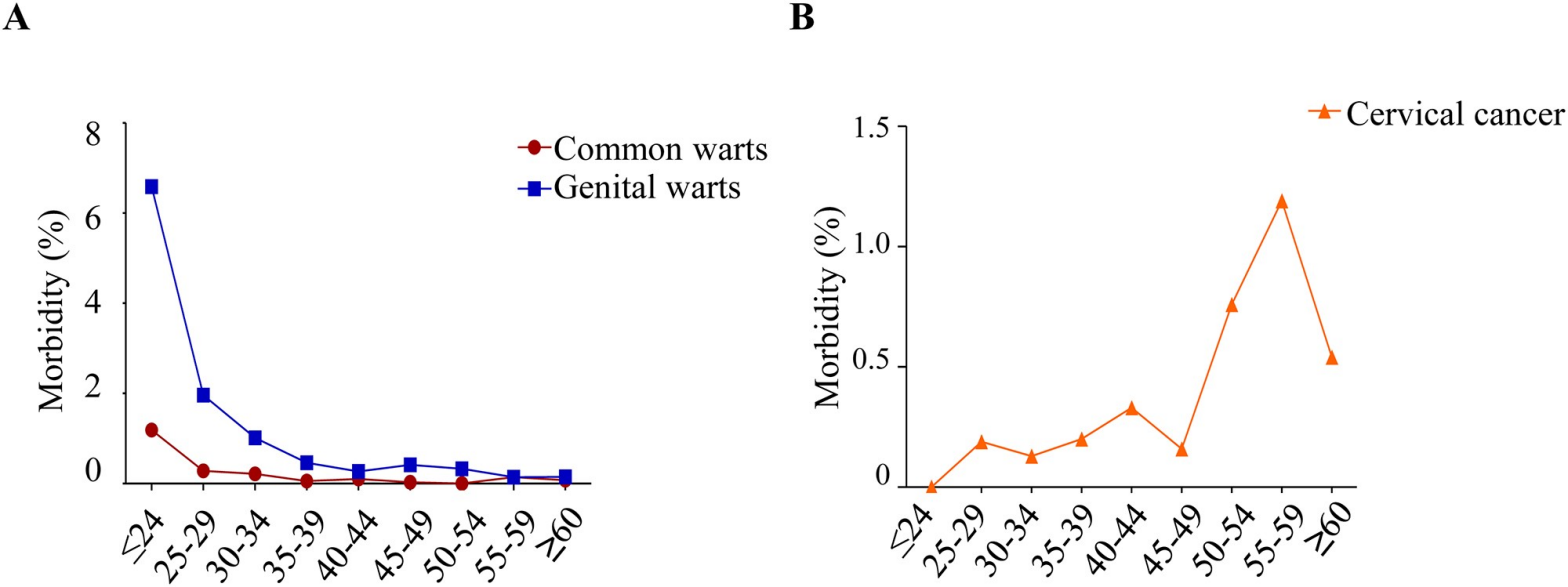
Overall age-related morbidity among women. (A)The morbidity of common warts and genital warts according to age. (B) The morbidity of cervical cancer according to age.

We investigated the morbidity of HPV-related diseases with a positive HPV genotyping, such as common warts, genital warts, CIN and cervical cancer. (Table 3 and Fig 2) Common warts, genital warts and cervical cancer showed an age-related morbidity (p < 0.001). (Table 3) The morbidity of common warts and genital warts decreased with age (p < 0.001). (Fig 2A) For people younger than 60, the prevalence of cervical cancer increased with age, and the prevalence showed a rapid decrease in people older than 60 years (p < 0.001). (Fig 2B).

**Table 3:** Overall age-related morbidity among women.

Age	N	Common warts (%)	Genital warts (%)	Cervicitis (%)	CIN (%)	Cervical cancer (%)
≤**24**	926	1.2	6.6	4.1	1.2	0.0
**25–29**	2141	0.3	2.0	4.6	1.4	0.2
**30–34**	3138	0.2	1.0	4.6	1.1	0.1
**35–39**	3490	0.1	0.5	3.6	1.1	0.2
**40–44**	3008	0.1	0.3	3.9	1.2	0.3
**45–49**	3125	0.0	0.4	3.7	1.3	0.2
**50–54**	2105	0.0	0.3	5.1	1.6	0.8
**55–59**	1423	0.1	0.1	4.8	1.6	1.2
**≥60**	1292	0.1	0.2	3.5	1.6	0.5

The morbidity of the HPV-related diseases with positive HPV genotyping.

### Genotype distribution of HPV types among women and men

**Fig 3:**
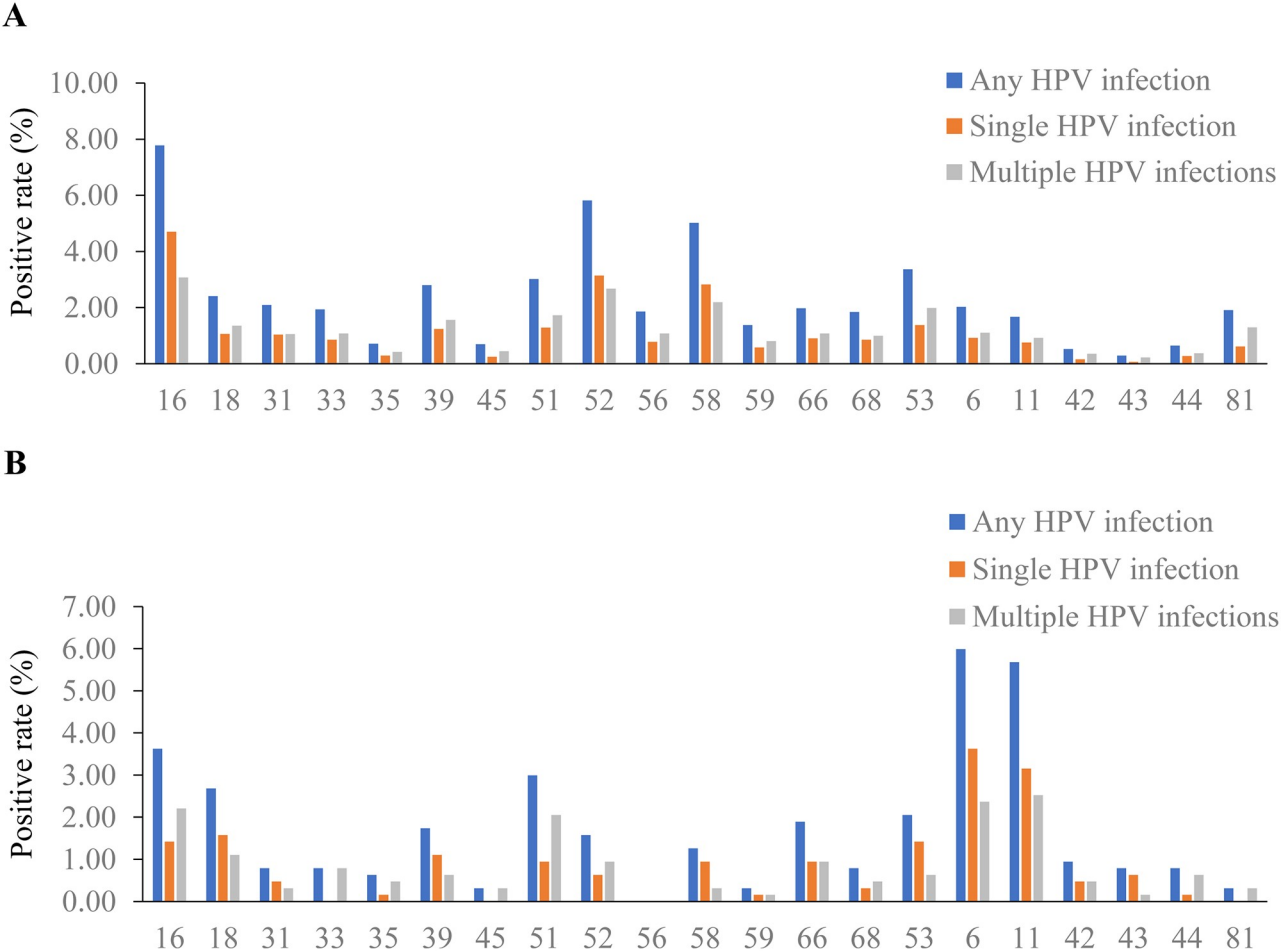
Genotype distribution of HPV types among women and men. (A) The genotype distribution of HPV types among women. (B) The genotype distribution of HPV types among men.

The genotype distributions of HPV types among women and men are presented in Fig 3A and 3B. Of the female individuals, HPV16 was detected as the most prevalent genotype (7. 8%), followed by HPV52 (5.8%), HPV58 (5.0%), HPV53 (3.4%), and HPV51 (3.0%) (Fig 3B). Among the LR-HPV types, HPV6 was the most prevalent genotype (2.0%), followed by HPV81 (1.9%) and HPV11 (1.7%). The prevalence of multiple HPV genotypes in all LR-HPV groups and most of the HR-HPV groups was higher than the single HPV genotype. Nevertheless, HPV16, HPV52, and HPV58 exhibited a higher prevalence as a single HPV genotype than that of multiple HPV genotypes. For multiple HPV infection, HPV16 (3.1%) was the most common genotype, followed by HPV52 (2.7%), HPV58 (2.2%), HPV53 (2.0%), and HPV51 (1.7%). Similarly, for single HPV infection, HPV16 (4.7%) was the most common genotype, followed by HPV52 (3.1%), HPV58 (2.8%), HPV53 (1.4%), and HPV51 (1.3%).

Of the male individuals, HPV6 was observed to be the most prevalent genotype (6.0%), followed by HPV11 (5.7%), HPV16 (3.6%), HPV18 (2.7%), and HPV51 (3.0%) (Fig 3A). Of the 5 most prevalent genotypes, the prevalence of single HPV infection with HPV6, HPV11, and HPV18 was higher than that of multiple HPV infections. For multiple HPV infection, HPV11 (2.5%) was the most commonly observed genotype, followed by HPV6 (2.4%), HPV16 (2.2%), HPV51 (2.1%), and HPV18 (1.1%). In contrast, HPV6 (3.6%) was the most common genotype for single HPV infection, followed by HPV11 (3.2%), HPV18 (1.6%), HPV16 (1.4%), and HPV53 (1.4%).

### HPV prevalence associated with cervical lesions

**Fig 4:**
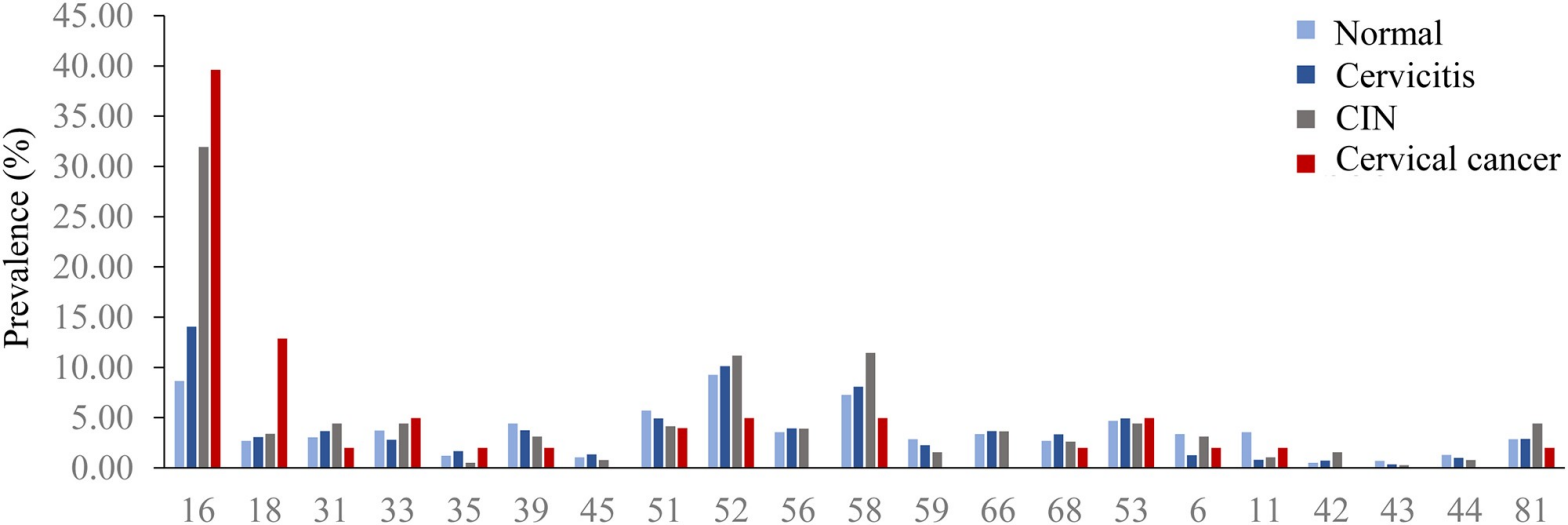
HPV prevalence associated with cervical lesions.

Among the 3142 females diagnosed with normal, cervicitis, cervical intraepithelial neoplasia (CIN) and cervical cancer, the prevalence of HPV was 51.4%, 57.5%, 69.4% and 70.3%, respectively. The prevalence of overall HPV increased with the severity of cervical lesions (p < 0.001). (Fig 4) The prevalence of HPV16 and HPV18 increased with the severity of cervical lesions (p < 0.001). HPV16 was the most prevalent genotype (8.7%) in normal tissue, followed by HPV52 (9.3%), HPV58 (7.3%), HPV51 (5.7%), and HPV53 (4.7%). For cervicitis, HPV16 (14.1%) was the most prevalent genotype, followed by HPV52 (10.2%), HPV58 (8.1%), HPV53 (4.9%), and HPV56 (3.9%). For CIN, HPV16 was the most prevalent genotype (32.0%), followed by HPV58 (11.4%), HPV52 (11.2%), and HPV31, 33, 53, and 81 (4.4%). For cervical cancer, HPV16 was the most commonly detected genotype (39.6%), followed by HPV18 (12.9%), and HPV33, 52, 53, 58 (5.0%).

### HPV prevalence associated with common warts and genital warts

**Fig 5:**
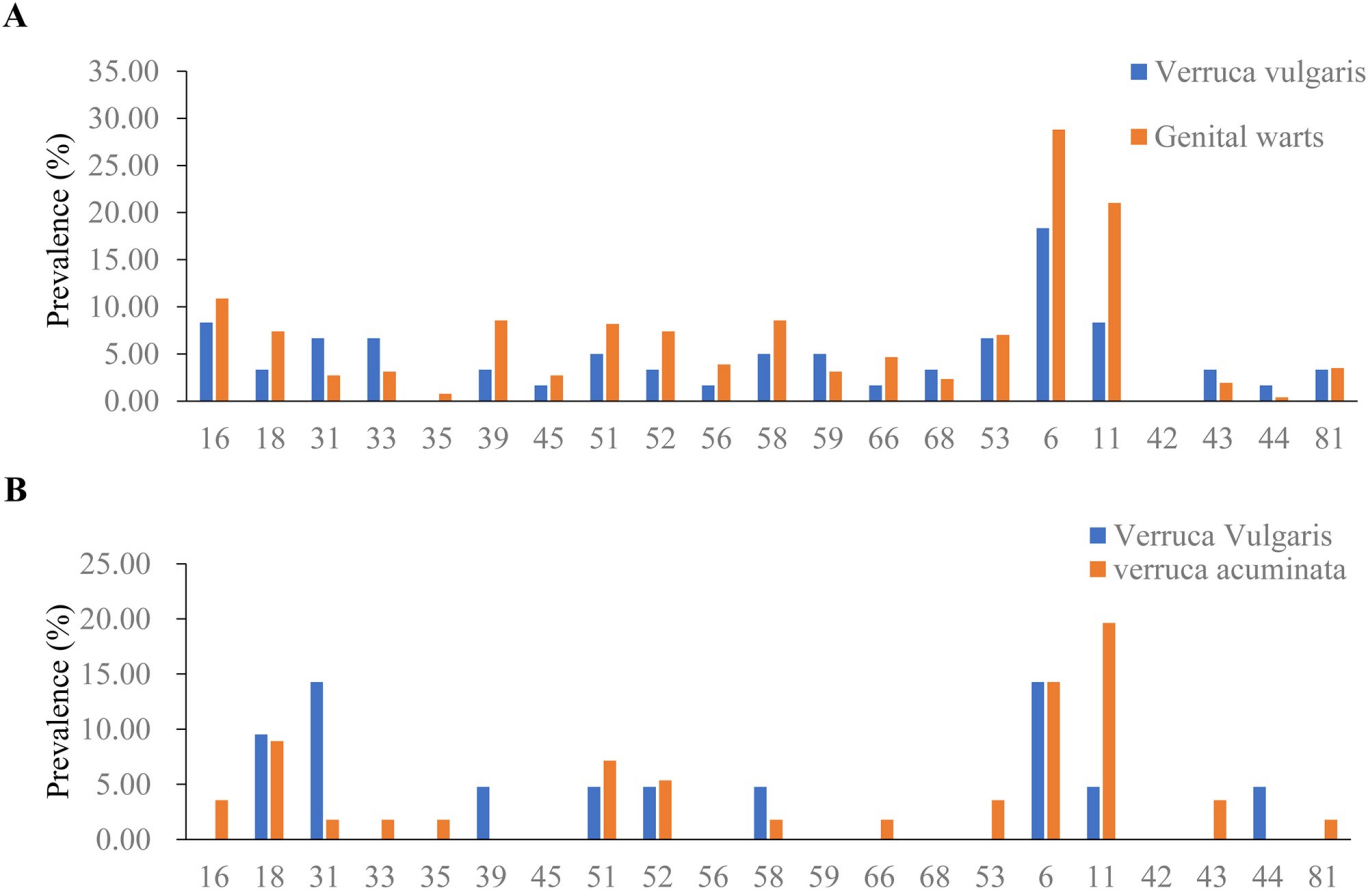
HPV prevalence associated with common warts and genital warts. (A) The HPV prevalence associated with common warts and genital warts in women. (B) The HPV prevalence associated with common warts and genital warts in men.

Among the 317 females with common warts and genital warts, the prevalence of HPV was 55.0% and 71.2%, respectively. (Table 4) For common warts, HPV6 (18.3%) was the most prevalent genotype, followed by HPV16 (8.6%), HPV11 (8.3%), HPV16 (8.3%), HPV33 (6.7%) and HPV56 (6.7%). For genital warts, HPV6 (28.8%) and HPV11 (21.0%) were the most prevalent genotypes, followed by HPV16 (10.9%), HPV39 (8.6%) and HPV58 (8.6%). (Fig 5A).

**Table 4:** HPV prevalence associated with common warts and genital warts.

		HR-HPV related	Only LR-HPV related	Total HPV
**Woman**	Common warts	36.7	18.3	55.0
	Genital warts	45.5	23.7	71.2
**Man**	Common warts	38.1	0.0	38.1
	Genital warts	28.6	21.4	53.6

The HR-HPV-related and LR-HPV-related prevalence of common warts and genital warts.

Among the 87 males with common warts and genital warts, the prevalence of HPV was 38.1% and 53.6%, respectively. (Table 4) For common warts, HPV6 and 31 (14.3%) were the most prevalent genotypes, followed by HPV18 (9.5%) and HPV11, 39, 51, 52, and 44 (4.8%). For genital warts, HPV11 (19.6%) was the most common genotype, followed by HPV6 (14.3%), HPV18 (8.9%), HPV51 (7.1%) and HPV52 (5.4%). (Fig 5B).

## Discussion

The present study provides large-scale data on the prevalence and genotype distribution of HPV in Jilin province, China. HPV infection rate varies between nations and regions. The overall prevalence of HPV (34.4%) among women in our study was higher than that in other regions of China, such as Shanghai (31.84%) [14], Shandong (28.4%) [2], Zhejiang (22.8%) [15], and Jiangxi (22.49%) [16]. Understanding the epidemiological characteristics of HPV infection is of great importance to the development of prevention and control strategies for HPV.

A bimodal pattern was shown in the prevalence of total HPV infection, single HR-HPV infection and multiple HPV infections among women, with a peak at younger group and a second peak at elderly group. This bimodal pattern is consistent with that found in other studies in China [15, 17, 18]. Similarly, the prevalence of specific HR-HPV genotypes, such as HPV16, 52, and 58, also exhibited a bimodal pattern. These results remind us to pay more attention to young women and old women in the prevention and control of HPV.

The first peak, where the LR-HPVs, such as HPV6 (9.0%) and HPV11 (8.9%), accounted for a crucial subset. The morbidity of common warts (1.2%) and verruca acuminate (6.6%) was higher in younger than in older age groups. This result may be related to earlier sexual activity and riskier sexual behaviors in younger age groups [19]. Considering the high prevalence of HPV infection, high morbidity of benign hyper-proliferative lesions, and the high transmission rate (60%) of HPVs produced from genital warts in this group [20], it is urgent to popularize the knowledge of HPV-related neoplasia and emphasize awareness of its causes, risk factors and care-seeking behavior in colleges and the community.

Cervical cancer screening consisting of cervical cytology and HPV genotyping is recommended as a regular physical examination beginning at 40 years old in northeast China. Considering the bimodal pattern of HPV infection, the age of cervical cancer screening should be advanced. However, most HPV infections in young people are temporary and naturally cleared by the immune system [21, 22], and cervical cancer is rarely observed among women younger than 20 years [23]. Excessive treatment of CIN 2 or CIN 3 among women younger than 21 years may increase the risk for adverse pregnancy outcomes [13]. It seems unnecessary to start screening before the age of 21 years, which is consistent with a previous study [24]. As recommended, cervical cancer screening should be performed every 3 years in women aged 21 to 29 years and may be performed with cervical cytology alone [13].

The second peak of total HPV prevalence appeared in 55 to 59 years old, before the period in which the morbidity of cervical cancer increased with age, which might reflect a naturally impaired immune response as a result of aging [17, 25]. After this age period, the HPV prevalence and the morbidity of cervical cancer exhibited a sharp decrease, which corresponds to the low estrogen levels of old women [26, 27].

The genotypic spectrum of HPV infection among women varies worldwide. In Asia, the most prevalent genotypes are HPV16 (2.6%), HPV52 (1.2%), HPV58 (1.0%), HPV18 (0.8%) and HPV56 (0.8%). In Europe, HPV16 (2.3%), HPV18 (0.7%), HPV31 (0.6%), HPV33 (0.4%) and HPV58 (0.4%) are the most prevalent genotypes [28]. In the Jilin province of China, HPV16 (7.8%), HPV52 (5.8%), HPV58 (5.0%), HPV53 (3.4%), and HPV51 (3.0%) are the most prevalent genotypes among women, which differs from that seen in other regions.

Cervicitis in this study was confirmed using cytological diagnosis without intraepithelial lesion or malignancy. Cervical intraepithelial neoplasia (CIN) is a group of precancerous lesions, and it is closely related to cervical cancer [29]. We analyzed the HPV prevalence associated with cervical lesions from normal to cervical cancer. Chronic cervicitis with persistent HPV infection was also associated with neoplasia, increased cellular epithelium turnover and enhanced genetic alterations conjointly with HPV infection [30–32]. The prevalence of overall HPV (especially HPV16 and 18) increased with the severity of cervical lesions. The 2 most prevalent genotypes in cervical cancer were the most common oncogenic types HPV16 and 18 regardless of the prevalence of HPV in normal, cervicitis and CIN. HPV52 and 58, which are overrepresented in Asia [33], were the most prevalent genotypes in all cervical lesions.

Nine-valent human papillomavirus (9vHPV) vaccine is widely used worldwide in preventing the infection of HPV16, 18, 6, 11, 31, 33, 45, 52 and 58 [34]. HPV53 and 51, which were found in 6.3% of HPV infection and 8.9% of cervical cancer in Jilin province of China, are not covered by the 9vHPV vaccine. An HPV16/18 vaccine and HPV16/18/6/11 vaccine showed some limited cross-protection against nonvaccine oncogenic HPV types, such as HPV31, 33, 45, and 51 [35–37]. Nonetheless, HPV prophylactic vaccines, including HPV53 and 51, may offer more sufficient protection for women in China.

HPV6 (6.0%), HPV11 (5.7%), HPV16 (3.6%), HPV18 (2.7%), and HPV51 (3.0%) were the most prevalent genotypes among men. Benign hyper-proliferative lesions, such as common warts and genital warts, whose pathogens are recognized to be LR-HPVs, but where HR-HPVs are also detected, are of great importance [38]. A total of 45.5% of females and 28.6% of males with genital warts were infected with HR-HPV genotypes, which is consistent with a study conducted in 2011 [39]. HR-HPV in genital warts of men contributed to HPV-related disease burden and affected their sex partner by increasing the rate of their cancer prevalence. In America, the number of HPV-associated noncervical cancers (especially HPV16 and 18), with an equal number of noncervical cancers among men and women, diagnosed annually approximates cervical cancers [9]. Given all of these factors, HPV infection in men and benign hyper-proliferative lesions should not be neglected. The vaccination of males holds great promise in reducing the burden of HPV-associated noncervical cancers. The Chinese government recently approved the 9vHPV vaccine, and it is difficult, due to its scarcity, to vaccinate males, but necessary.

## Conclusion

The HPV genotypic spectrum in Jilin province, where non-vaccine-covered HPV53 and 51 were prevalent, exhibited an age- and cervical lesion-specific pattern, which implicated the future HPV-related malignancy burden. HPV infection in men and benign hyper-proliferative lesions should not be neglected. The large-scale data on the prevalence and genotype distribution of HPV provided in this study provides guidance for HPV prophylactic vaccines and cervical cancer screening.

## Supporting information

S1 Data (XLSX)
